# Recruiting medical groups for research: relationships, reputation, requirements, rewards, reciprocity, resolution, and respect

**DOI:** 10.1186/1748-5908-1-25

**Published:** 2006-10-26

**Authors:** Leif I Solberg

**Affiliations:** 1 HealthPartners Research Foundation, PO Box 1524, MS#21111R, Minneapolis MN 55440-1524, USA

## Abstract

**Background:**

In order to conduct good implementation science research, it will be necessary to recruit and obtain good cooperation and comprehensive information from complete medical practice organizations. The goal of this paper is to report an effective example of such a recruitment effort for a study of the organizational aspects of depression care quality.

**Methods:**

There were 41 medical groups in the Minnesota region that were eligible for participation in the study because they had sufficient numbers of patients with depression. We documented the steps required to both recruit their participation in this study and obtain their completion of two questionnaire surveys and two telephone interviews.

**Results:**

All 41 medical groups agreed to participate and consented to our use of confidential data about their care quality. In addition, all 82 medical directors and quality improvement coordinators completed the necessary questionnaires and interviews. The key factors explaining this success can be summarized as the seven R's: Relationships, Reputation, Requirements, Rewards, Reciprocity, Resolution, and Respect.

**Conclusion:**

While all studies will not have all of these factors in such good alignment, attention to them may be important to other efforts to add to our knowledge of implementation science.

## Background

There is an extensive literature of studies and recommendations about methods to enhance the response rate of physicians to research surveys [1-3], and a few studies of strategies to recruit physicians to participate in research projects [4,5]. However, there is very little information about methods for recruiting entire medical group practices for research studies of the organizational aspects of quality improvement. Studies by McBride, et al and Carey, et al do provide some guidance, but an overall framework and details for such recruitment remain to be elaborated [6,7]. Understanding how to recruit complete group practices has become increasingly important as studies of how to improve the quality of care have shifted their focus from the behavior of individual physicians to the environment in which they work [8-13]. In fact, this kind of recruitment has become such a necessity for good implementation science that many of these studies can't be done effectively without involvement of a representative cross-section of entire eligible medical practices.

Recently, we were so successful in recruiting and sustaining the effective participation of many medical groups for such a research project that it seemed important to summarize the lessons learned. The research project's goal was to analyze the relationship between the organizational and environmental factors of entire medical groups, as well as their rates of performance on a common measure of quality of care for patients with depression. In order to make this analysis valid, we needed to recruit as many as possible of the medical groups in Minnesota that had such performance data available in a standardized format and process from a community organization that collected and publicly reported such rates. This paper's purpose is to document the approach and efforts involved in that recruitment and to identify the factors that appeared to be important for that success. While there are an increasing number of studies of organizational factor relationship to care quality, virtually none of those studies provide any detailed information about the recruitment process used or the types of barriers that need to be overcome. For example, the growing series of NSPO (National Study of Physician Organizations) studies that recruited 1040 large medical groups simply reported a preliminary phone call to verify eligibility for a subsequent phone interview, without reporting who made or received the calls or what was involved [14].

The methods chosen for this recruitment borrowed, in part, from the cited literature, but mostly reflect our own mostly unpublished experience over the past 15 years with recruitment of physicians and practices for many different research projects. For the most part, the literature and published evidence is simply insufficient to allow a novice to come up with a recruitment plan with any chance of success. What was clear from our experience was the importance of using a physician to recruit physicians and of having credible answers for the immediate, nearly universal questions about how much time and effort would be involved, whether or not it would interfere with patient care, and what value would result for themselves and others.

## Methods

### Context

Primary medical care in Minnesota is provided mainly by relatively large medical groups to the point of there being relatively few medical groups limited to a single geographic site and virtually no remaining 1–2 physician practices. These medical group practices have often been formed through purchase by a health plan, hospital, or large multi-specialty group, but there also are many large, single-specialty primary care medical groups that are the result of mergers. Thus, most groups have multiple practice sites or clinics as well as an identifiable medical director and significant administrative infrastructure that provide common systems across sites and physicians. Over the past three years, a public reporting organization called Minnesota Community Measurement (MN CM) has developed out of a collaboration among all the health plans in the state [15]. This organization collects, analyzes and publicly reports comparative performance data on a variety of quality measures that resemble HEDIS^® ^(Health Plan Employer Data and Information Set) measures [16]. One of those measures is called Continuation Phase Treatment, which records the proportion of depressed patients who are started on a new antidepressant medication and stay on it for 180 days [17]. Only 41 medical groups in Minnesota and bordering areas have enough patients identifiable through a combination of multiple health plan data sets to permit accurate measurement of this rate. These 41 groups collectively provide most of the primary care in the region and were the target of this recruitment effort.

### Research participation requirements

In order to meet the goal of this research project, participating medical groups had to agree to complete all of the following requirements:

1. Signed consent for the research project to obtain all of their data from MN CM, including the data underlying the calculated performance rates;

2. Completion by the medical director (and other staff as needed) of a 180-item questionnaire asking detailed questions about the presence of a wide variety of organizational systems for providing chronic disease care, as well as descriptive data about the medical group [18];

3. Participation by the medical director in a 15–30 minute telephone interview asking about that medical group's priority for improving depression care and about the specific actions taken in that regard, as well as perceptions of the barriers and facilitators for such improvement;

4. Completion by the staff person most familiar with the quality improvement (QI) efforts of the medical group (usually the QI Coordinator) of a 40-item questionnaire asking about organizational factors and improvement strategies used in that group for depression improvement; and

5. Participation by that same staff person in a 15–30 minute telephone interview asking more open-ended questions about that group's depression care improvement efforts and perceived barriers and facilitators for improvement.

### Research participation rewards

1. $100 to the medical group as helping to defray the time costs and as a thank-you for participating, although this was never mentioned by leaders as an important consideration;

2. Promise of receiving the results of the research – both overall lessons and their own data in comparison to the average for all participating medical groups; and

3. Promise of acknowledgement of their contributions in any publications.

In addition, participants were assured that all of their data would be kept completely confidential, reported only in anonymous aggregation. They also were assured that we would make every effort to minimize intrusion in the time or operational work of any respondents.

### Recruitment process

1. An initial letter and project brochure was mailed to the medical director of each eligible group that described the project along with the above requirements and rewards, and advised that the principal investigator (LS) would be calling in the next few weeks.

2. The principal investigator recruiter made as many telephone calls as needed to reach each medical director. During the call, he answered any questions, asked about willingness to participate, and arranged a specific follow-up contact plan.

3. The recruiter then made as many follow-up telephone calls as necessary until either a refusal or verbal agreement to participate was obtained. At the time of an agreement, he obtained the name and contact information for the quality improvement staff.

4. A consent letter and the survey were mailed to the director, re-specifying the research requirements and rewards and asking for signed consent and return of a completed survey.

5. At one and a half weeks after this mailing, the recruiter sent an e mail to the director as a reminder, and then made as many telephone calls as necessary until the signed consent letter and completed survey were received.

An ACCESS tracking data base was developed to facilitate tracking and reminders to provide timely monitoring and follow-up of the recruitment steps and the arrangements and follow-up for the surveys and interviews. This database also provided the information for this report. Reflecting on the entire process and on many similar recruitment efforts in the past led the author to summarize his impression of the main factors that seemed to be associated with success. Listing these factors led to a realization that each factor name or a synonym began with R, making it possible to create a useful memory device that led to the title of this paper. All steps in the process were reviewed, approved and monitored by an IRB.

## Results

Every one of the 41 eligible medical groups agreed to participate in this study, and all of the required consent forms, surveys and interviews were successfully completed for 100% participation and compliance. Table 1 provides a summary description of these groups. It confirms that these groups were mostly large with multiple sites.

**Table 1:** Description of Participating Medical Groups (N = 41)

**Characteristic**	**N**	**%**
Number of physicians:		
<10	1	2
10–39	10	24
40–99	13	32
100–199	8	20
200–2000	9	22
Number of practice sites:		
1	2	5
2–5	17	41
6–15	11	27
>15	11	27
Type of practice:		
Primary care	15	36
Multi-specialty	26	63
Ownership:		
Health plan	3	7
Hospital	12	29
Physicians	19	46
Other	7	17
Patient visits/week/group	4800	800–50,000
Commercial insurance	61%	28–87%
Medicare	20%	5–40%
Medical Assistance	9%	0–54%
No insurance	3%	0–16%

In Table 2, we document the number of calls, discussions and days required to complete the recruitment process and follow-up on the medical director's consent and survey. It shows that for 19 of the medical groups, only 1–2 calls and a single discussion were required to obtain agreement to participate. All but three of these directors returned their consent forms and surveys promptly as well, either requiring no follow-up calls (13) or only a single call (3). On the other hand, 14 directors required three or more calls, two-five discussions, and more than two weeks (except one of eight days) to recruit them; nine because they needed to get the approval of other people or some management group. Of these 14, nine also required multiple phone calls and more than two weeks after the first follow-up phone call to return their surveys.

**Table 2:** Contact and Time Requirements for Recruitment and Survey Return (n = 41)

**Action**	**Recruitment**	**Survey Return**
Number of calls made:		
Total (mean)	154 (3.8)	89 (2.2)
0	-	19
1	8	7
2	13	1
3–7	16	12
>7	4	2
Number of phone discussions:		
Total (mean)	84 (2.0)	49 (1.2)
0	-	20
1	24	9
2–3	10	8
4–6	7	4
Days from first call to last:		
Mean	13.5	13.9
0	-	19
1–7	22	7
8–14	4	1
15–21	6	3
22–28	3	0
>28	4	11
Recruitment required approval from others	10	N/A
Number of days if approval required: range (mean)	13–97 (36)	N/A

Each of the following R-factors appeared to play an important role in obtaining the participation and completion of data collection from this varied group of medical directors:

### Relationship

The recruiter had been in the local medical community for 30 years and already knew about half of the eligible medical directors from previous contacts. His status as a physician-peer also clearly helped to develop a relationship, if none existed before. Also, the opportunity to speak directly to the medical director was facilitated greatly by the widely accepted custom of providing immediate access to a physician when another physician calls.

### Reputation

The recruiter was not only known to many directors, but had established a reputation for doing practical research and being very interested in quality improvement. He also was known to not abuse relationships or information.

### Requirements

While the requirements for research participation were not minimal, they did not require large investments of time. More importantly, they could be met without requiring any time from other busy physicians in the group.

### Rewards

Although the financial incentive was minimal, it at least provided some recognition of the fact that a donation of valuable time was being requested. More important was the promised information about their own group's approach relative to competing groups, and the lessons about which strategies might be most valuable for improving depression. Unlike diabetes care, there is widespread uncertainty about how to improve care for this problem.

### Reciprocity

Although similar to the concepts above, the explicit recognition that there is a mutual obligation that is negotiated has seemed key to the collaborative nature of the study: "Here is what I will do for you, and this is what I hope you will do in return."

### Resolution

What is really meant here is persistence – the willingness to repeatedly make contact efforts until the right person is reached for interaction and an agreement can be reached, while walking the fine line between nagging and leaving things as is. Table 2 provides evidence for this.

### Respect

This really sums up all of the above. Because the recruiter genuinely respected the subjects, their work, and their constraints, he never took them or their participation for granted.

## Discussion

While the seven factors identified here may come as no surprise to anyone who has faced the task of recruiting entire medical practices for research studies, they have not previously been either explicitly identified as a group or demonstrated to be so successfully combined in one research study. In fact, most of the prominent studies of organizational behavior do not report much information on the methods used to recruit participant organizations. For example, one of the most well known of such recent studies – the National Survey of Physician Organizations (NSPO) – only reported the organizational response rate to its survey (70%), but nothing about the methods involved in obtaining agreement to participate [14]. This study was limited to medical groups or IPAs (Independent Practice Associations) with more than 20 physicians, and found that medical groups were less likely than IPAs to respond to the survey (66% vs. 79%, P < .001).

Another study in the Minnesota region obtained a 90% response rate to surveys of the medical director and administrator of 172 individual clinics about their organizational structure [19]. Perhaps this means that there is an additional R factor for *Region *of the country, but the same research group more recently obtained only a 71% response rate from the administrators of 127 group practices for a survey about practice structure [9]. Even a strongly hierarchical medical care organization such as the Veteran's Health Administration wasn't able to obtain high response rates to surveys of VA medical center quality managers and primary care administrators about their efforts to improve quality of care [20]. Although the latter article at least reported some of the details of their survey methods, none of these or other studies of care delivery organizations provide enough information about their recruitment and survey methods to allow others to know, for example, whether any of the R-factors reported here were used.

The few studies reporting on recruitment of group practices note the benefit of recruitment through the group's physician leader or medical director, as was done in this study. McBride recruited 65% of eligible practices in the Midwest by dealing with the practice leader, but 54% were recruited through mailings to individual physicians [6]. He recommended phone calls from study physicians to practice medical directors followed by recruitment meetings at the practice site. Kottke also compared different methods, finding that only 6% of individual family physicians and 2.7% of internists and cardiologists recruited by mail, with a follow-up phone call if interested, ended up participating in a smoking cessation trial [4]. In both cases, the project had been endorsed by the respective local professional associations. However, when 11 groups were approached through their medical director on behalf of a local health plan, all 11 groups participated and a mail survey of physicians in these groups achieved an 86% response rate. Again, practice informational meetings were held to familiarize all personnel with the project. Although neither of these reports specifically discussed the R-factors noted in this study, most of them appear to have been involved, at least to some extent.

Two other reports provide some information to corroborate these observations and recommendations. Carey, et al [7] report on a variety of aspects of conducting research in community practices in North Carolina and note several components that contributed to success:

1. "Direct recruitment of clinicians by clinicians,

2. Ongoing personal contact to maintain the relationship, and

3. Recognition of the value of the community clinicians' time."

Ganz, et al describe recruitment of what they call 'provider organizations' in California, although most of these groups appear to have been much less integrated than the medical groups described here [21]. They recruited 71% of 174 provider organizations for a medical director survey and 71% of a subset of 51 for a randomized trial, reporting an average of five calls and 37 days to get initial agreement to participate in the trial (compared to two and 14 in this study).

Our experience and the literature suggest that it is very important to have a physician recruiter for physician subjects. Researchers without that degree would be well-advised to partner with a physician to do this recruitment, ideally one with a good local reputation and established relationships. Lacking those R's, however, an unknown physician will at least facilitate access and credibility.

This report of an apparently successful approach to recruiting entire medical groups for a research study does have some limitations. The practice of medicine in this region is unusually collective, both in having most physicians in relatively large groups and in having a relatively high degree of integration of the practices within most groups. There also may be a greater sense of community cooperation here. However, other than being of sufficient size to have enough depressed patients for this study, there is nothing about these 41 medical groups that would make them more responsive to recruitment for this study. Even "small" medical groups in this region have a designated medical director as a focus for recruitment and study coordination, perhaps in part because the high managed care penetration in this region virtually requires such an organized management. This local characteristic of medical groups may affect generalizability to some other regions with mostly small practices, although we find that even in such areas there is usually at least an informal physician leader, often the practice founder. *Requirement *of time for participation will be a similar issue for all groups, large or small, because of the pressure on any primary care practice and its leaders.

A greater limitation for generalizability may be the reputation and pre-existing relationship of the recruiting physician with many of the medical group leaders. The impending appearance of pay-for-performance may have contributed to an increased willingness of medical groups to participate in studies that will inform those efforts, but there was nothing specific about this study or the measures used that were tied directly to such efforts in the minds of recruitment subjects.

A discussion of practice recruitment for participation in research would not be complete without mentioning practice-based research networks (PBRNs). These existing aggregations of physicians and/or practices were developed in the 70's and have increased to the point of there being 111 identifiable PBRNs throughout the U.S. in 2003 [22]. According to a report of a survey of 87 PBRN's from the AHRQ-funded PBRN Resource Center at the University of Indiana, they contained 2,724 practices caring for 14.7 million patients in 44 states and Puerto Rico. While these networks represent a valuable resource, they are usually small (average size of 4.7 physicians per practice), and many began or continue as aggregations of research-interested individual physicians rather than whole medical groups. They also may not fit geographically or demographically with the needs of many research studies, and they may not be willing or able to participate. Finally, this study is an example of a project that could not have used a PBRN, since eligibility required that they have outcome data in a public accountability set, and most were not members of the local PBRN.

## Conclusion

Whether one works through a PBRN or recruits needed practices independently for an implementation research project, the seven R-factors seem to be important. They are not only needed for recruitment, but also for the good cooperation and maintenance that are necessary throughout a research study. They also are likely needed for working with practices in such a way that the lessons of the research are capable of being implemented in the participating practices, and that is increasingly as important as doing the research itself.
